# Association Between Increased Posterior Tibial Slope and Isolated Meniscal Tears in Patients Aged 18–30 Years

**DOI:** 10.3390/jcm15156054

**Published:** 2026-08-04

**Authors:** Musa Alperen Bilgin, Burcin Karslı, Vahap Kurt, Nevzat Gönder

**Affiliations:** 1 Department of Orthopaedics and Traumatology, Aksaray Training and Research Hospital, Aksaray 68000, Türkiye; alpperren@gmail.com; 2 Department of Orthopaedics and Traumatology, Faculty of Medicine, Sanko University, Gaziantep 27090, Türkiye; drkarsli@gmail.com; 3 Department of Orthopaedics and Traumatology, Abdülkadir Yüksel State Hospital, Gaziantep 27000, Türkiye; vahapkurt@hotmail.com; 4 Department of Orthopaedics and Traumatology, Faculty of Medicine, Gaziantep University, Gaziantep 27310, Türkiye

**Keywords:** posterior tibial slope, meniscus, meniscal tears, knee, radiography

## Abstract

**Background/Objectives:** Meniscal tears frequently occur during athletic activities and are often associated with anterior cruciate ligament injuries. The purpose of this study was to compare the posterior tibial slope (PTS) in young adults undergoing arthroscopic surgery for isolated meniscal tears with that of a control group, and to investigate its association with meniscal tears. **Methods:** This retrospective, single-centre, controlled study included 190 patients aged 18–30 years who presented with unilateral knee pain and underwent knee magnetic resonance imaging (MRI) between 2015 and 2022. Patients with an isolated medial meniscal tear (Group A, n = 67) or an isolated lateral meniscal tear (Group B, n = 31), confirmed on MRI and directly with arthroscopy, were compared with a symptomatic control group who presented with knee pain but had no meniscal or anterior cruciate ligament (ACL) tear on MRI (Group C, n = 92). Medial and lateral PTS angles were measured on pre-existing standing lateral knee radiographs. Interobserver and intraobserver reliability were quantified using intraclass correlation coefficients (ICCs). Group comparisons, receiver operating characteristic (ROC) analysis with the Youden index, and multivariable binary logistic regression adjusted for age, sex and side were performed. **Results:** Both medial and lateral PTS angles were significantly higher in Groups A and B than in the control group (*p* < 0.05). ROC analysis identified a cut-off of 9.2° for the medial PTS in the medial-tear group (AUC: 0.756, 95% CI: 0.68–0.83; sensitivity: 64.18%, specificity: 80.43%) and 9.9° for the lateral PTS in the lateral-tear group (AUC: 0.709, 95% CI: 0.60–0.82; sensitivity: 58.06%, specificity: 81.52%). The association persisted after adjustment for age, sex and side (medial slope in medial tears adjusted OR: 1.36 per 1°, 95% CI: 1.19–1.55, *p* < 0.001; lateral slope in lateral tears adjusted OR: 1.25 per 1°, 95% CI: 1.08–1.44, *p* = 0.003). **Conclusions:** In this retrospective cohort of young adults, an increased posterior tibial slope was associated with isolated meniscal tears. A medial PTS greater than 9.2° and a lateral PTS greater than 9.9° were associated with medial and lateral meniscal tears, respectively. Given the modest sensitivity and the observational design, PTS should be regarded as a supportive morphological marker rather than a stand-alone diagnostic metric, and a causal relationship cannot be inferred based on the results of this study. Because discrimination was only fair (AUC 0.693–0.756) and sensitivity limited (54.8–64.2%), these cut-offs are not clinically applicable decision thresholds and should be used only in conjunction with clinical assessment and MRI.

## 1. Introduction

Meniscal tears frequently occur during athletic activities and are often accompanied by anterior cruciate ligament (ACL) injuries. Untreated meniscal tears can lead to permanent functional impairment and disrupt load distribution across the tibiofemoral joint, contributing to accelerated articular cartilage wear and the early development of post-traumatic osteoarthritis [1,2].

Risk factors for meniscal tears are generally divided into extrinsic and intrinsic categories. Extrinsic factors include neuromuscular forces, specific injury mechanisms, delayed surgery and untreated ligament injuries [3,4,5]. Intrinsic factors include age, sex, lower-extremity alignment, body mass index (BMI) and the geometric characteristics of the tibial plateau [6,7,8]. Among these anatomical variables, the posterior tibial slope (PTS) can be readily quantified and may help orthopaedic surgeons in both diagnosis and treatment planning when a meniscal tear is suspected.

An increased PTS alters knee biomechanics by augmenting the anteriorly directed shear force acting on the tibial plateau, which increases anterior tibial translation and the mechanical demand placed on the intra-articular soft tissues [9,10,11,12]. Although much of this evidence originates from ACL-deficient knees, the same biomechanical mechanism is expected to raise the load borne by the menisci and thereby predispose to meniscal injury [9]. Accordingly, several authors have reported an association between an increased tibial slope and meniscal pathology [8,9,13,14,15].

However, the existing evidence has three important limitations. First, most studies have examined PTS in the setting of concomitant ACL insufficiency, so the independent relationship between PTS and isolated meniscal tears remains poorly defined. Second, the majority of reports have relied on magnetic resonance imaging (MRI) or have included heterogeneous age ranges, whereas PTS tends to flatten with advancing age [16], which may confound comparisons drawn from mixed-age cohorts. Third, no clear consensus has emerged regarding side-specific (medial vs. lateral) slope thresholds for meniscal tears [17,18]. Meniscal injuries can also occur through non-contact mechanisms, underscoring the potential contribution of individual bony morphology.

We therefore focused on a homogeneous cohort of young adults aged 18–30 years—the age band with the highest participation in pivoting and high-demand athletic activities and in whom the PTS is relatively high and stable—minimising age-related confounding. The novelty of the present study lies in the combination of (i) an exclusively young-adult population; (ii) isolated, arthroscopically confirmed medial or lateral meniscal tears; and (iii) side-specific medial (MPTS) and lateral (LPTS) slope measurements obtained from inexpensive, widely available plain radiographs. Accordingly, this study aims to examine, in detail, the relationship between meniscal tears and the posterior tibial slope by comparing the MPTS and LPTS of patients with meniscal tears and control participants [19].

## 2. Materials and Methods

### 2.1. Study Design

**Figure 1:**
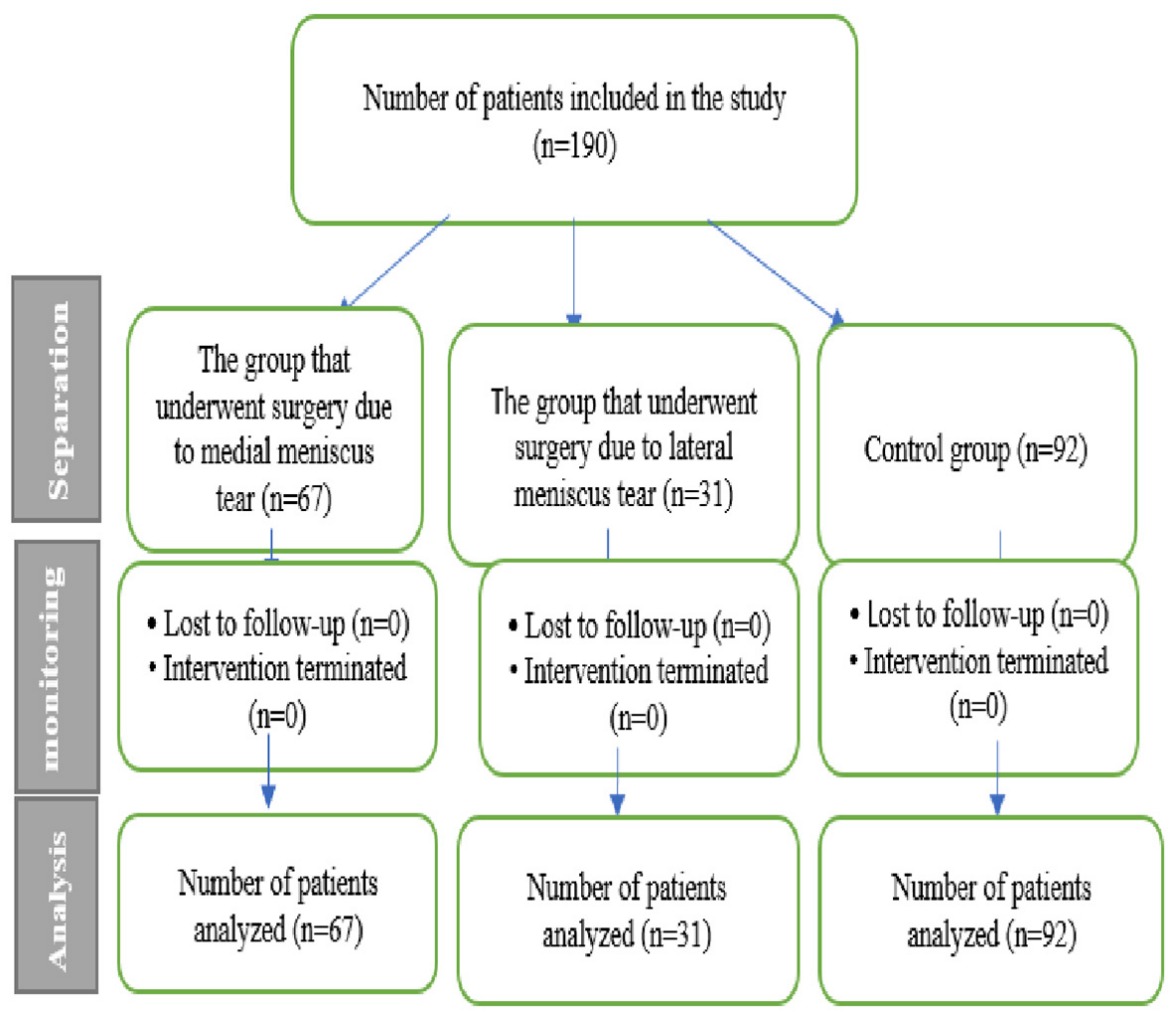
Flow diagram of patient selection and group allocation. Patients aged 18–30 years who presented to a single centre with unilateral knee pain and underwent knee magnetic resonance imaging (MRI) between 2015 and 2022 were screened. Patients were excluded if they had bilateral knee complaints, a concurrent ligament injury, a severe cartilage lesion, a discoid meniscus, a concomitant bone fracture, or technically inadequate radiographs or incomplete records. The final study population comprised 190 knees allocated to three groups: Group A, isolated medial meniscal tear identified on MRI and confirmed at arthroscopy (n = 67); Group B, isolated lateral meniscal tear identified on MRI and confirmed at arthroscopy (n = 31); and Group C, symptomatic controls in whom MRI demonstrated no meniscal or anterior cruciate ligament (ACL) tear (n = 92). All three groups were required to have technically adequate standing anteroposterior and lateral knee radiographs available for slope measurement. ACL, anterior cruciate ligament; MRI, magnetic resonance imaging.

This retrospective, single-centre, controlled study included 190 patients aged 18–30 years who presented to the Department of Orthopaedics and Traumatology, Gaziantep University Faculty of Medicine, with unilateral knee pain and underwent MRI between 2015 and 2022 (Figure 1). Because the diagnostic and control cohorts served different purposes, the eligibility criteria are reported separately below.

Inclusion criteria for meniscal-tear groups (Groups A and B): Patients aged 18–30 years presenting with unilateral knee pain; an isolated medial (Group A) or lateral (Group B) meniscal tear identified on MRI and subsequently confirmed by arthroscopy; and technically adequate full-length standing anteroposterior and lateral knee radiographs available in the hospital database.

Inclusion criteria for control group (Group C): Age-eligible patients who presented with knee pain during the same period and in whom MRI demonstrated no meniscal or ACL tear (i.e., symptomatic controls with a normal meniscal/ligamentous MRI), with radiographs of equivalent technical quality.

Exclusion criteria (all groups): Age under 18 or over 30 years; bilateral knee complaints; a concurrent ligament injury; a severe cartilage lesion; a discoid meniscus; a concomitant bone fracture; or inadequate radiographs or incomplete records.

The PTS was measured on the standing lateral knee radiographs that had been obtained at the initial presentation, using the method described by Hohmann et al. [20]. Because the PTS is a fixed osseous anatomical parameter that is not altered by meniscal surgery, arthroscopy served only to confirm the presence and location of the tear and did not influence the radiographic measurements. Measurements were performed by an investigator blinded to group allocation, and were repeated three times and averaged.

This study was performed in accordance with the principles of the Declaration of Helsinki and was approved by the Gaziantep University Non-Interventional Research Ethics Committee (2022; No. 2022/214). Because of the retrospective design and the use of anonymised, routinely collected clinical data, the ethics committee waived the requirement for written informed consent.

An a priori power analysis (primary outcome: between-group difference in PTS; two-sided α = 0.05; power 1 − β = 0.80; anticipated large effect based on prior slope studies [19,21]) indicated that a minimum of 17 participants per group was required. Groups of comparable size were assembled to preserve the balance between the tear and control cohorts.

### 2.2. Study Groups

Participants were divided into three groups: Group A (patients with an isolated medial meniscal tear), Group B (patients with an isolated lateral meniscal tear) and Group C (symptomatic controls without a meniscal or ACL tear on MRI). Sex, tear side, tear type and the medial and lateral PTS angles were compared between the groups. The sensitivity and specificity of the PTS values were evaluated using ROC analysis.

### 2.3. Posterior Tibial Slope Angle

**Figure 2:**
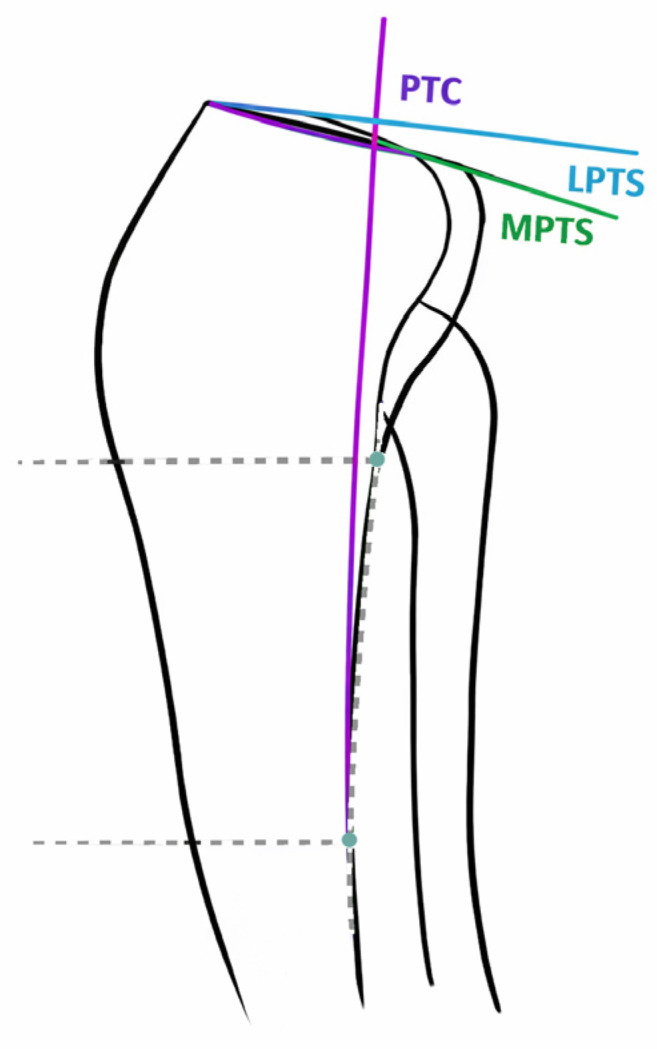
Schematic illustration of the technique used to measure the medial (MPTS) and lateral (LPTS) posterior tibial slope angles on the lateral knee radiograph, according to the method described by Hohmann et al. [20]. The image is a schematic line drawing of the measurement technique and is not a patient radiograph. Throughout the figure, lines that serve only to locate reference points, and are not themselves measurement lines, are drawn as dashed grey lines. Two transverse construction lines are first drawn across the proximal tibia at two separate levels; the point at which each transverse line meets the posterior tibial cortex is marked by a teal circle, and a straight line is then drawn along the posterior tibial cortex between these two reference points to give the posterior tibial cortex (PTC) axis (solid purple), which serves as the longitudinal reference axis of the tibia. The contour of the posterior cortex itself is traced as a dashed grey line along the tibial shaft. The PTC axis is extended proximally beyond the joint line. Two measurement lines are then drawn along the articular surface of the proximal tibia, each connecting the anterior and posterior margins of the highest point of the respective plateau: the medial plateau line (solid green, labelled MPTS) and the lateral plateau line (solid blue, labelled LPTS). The posterior tibial slope of each compartment is the angle between the corresponding plateau line and the line perpendicular to the PTC axis, that is, the complement (90° − θ) of the angle θ subtended between the extended PTC axis and that plateau line at their point of intersection. A plateau surface inclined downwards in the posterior direction relative to the perpendicular yields a positive slope value. The identical construction was applied separately to the medial and the lateral plateau in every knee, giving the MPTS and the LPTS, respectively. LPTS, lateral posterior tibial slope; MPTS, medial posterior tibial slope; PTC, posterior tibial cortex.

The PTS angle was measured on the standing lateral radiographs of all participants using the method first described by Hohmann and colleagues [20]. The medial and lateral PTS angles were defined as the angle between (i) a line perpendicular to the posterior tibial cortex (PTC) and (ii) the line connecting the anterior and posterior margins of the highest point of the respective tibial plateau (Figure 2). To standardise nomenclature, the medial and lateral posterior tibial slope angles are hereafter abbreviated as MPTS and LPTS, respectively.

All measurements were performed by a single orthopaedic surgeon blinded to group allocation and were repeated on three separate occasions, with the mean value used for analysis (intraobserver reliability). To assess interobserver reliability, a second orthopaedic surgeon independently re-measured a random subset of 40 knees. Agreement was quantified using the intraclass correlation coefficient (ICC; two-way random-effects model, absolute agreement). The intraobserver ICC was 0.94 (95% CI: 0.91–0.96) for MPTS and 0.92 (95% CI: 0.88–0.95) for LPTS, while the interobserver ICC was 0.89 (95% CI: 0.83–0.93) for MPTS and 0.87 (95% CI: 0.80–0.92) for LPTS, indicating excellent measurement reliability.

### 2.4. Statistical Analysis

Numerical variables are presented as the mean ± standard deviation and categorical variables as the frequency and percentage. The normality of the angle measurements was verified with the Shapiro–Wilk test. Between-group differences in MPTS and LPTS were assessed using one-way analysis of variance (ANOVA), followed by the Tukey HSD post hoc test for pairwise comparisons; for each pairwise comparison, we report the mean difference, its 95% confidence interval (CI) and the standardised effect size (Cohen’s d). Medial versus lateral slope within a group was compared using the paired-samples *t*-test. Categorical variables were compared using the chi-square test. Diagnostic performance was evaluated by ROC analysis; the area under the curve (AUC) with its 95% CI is reported, and optimal cut-off values were selected using the Youden index. To determine whether the PTS was independently associated with a meniscal tear, multivariable binary logistic regression models were constructed (medial tear vs. control and lateral tear vs. control), adjusting for age, sex and side, and the adjusted odds ratios (ORs) and 95% CIs are reported. Analyses were performed in IBM SPSS Statistics v22.0, with a two-sided *p* < 0.05 considered significant.

## 3. Results

A total of 190 patients who met the inclusion criteria were analysed; 125 patients (65.79%) were male and 65 (34.21%) were female, and 97 (51.05%) had right-knee complaints and 93 (48.95%) had left-knee complaints. The MPTS and LPTS were evaluated for medial meniscal and lateral meniscal tears, respectively. The demographic and clinical characteristics of the three groups are summarised in Table 1.

Group A comprised 67 patients (35.26%) who had a medial meniscal tear, Group B comprised 31 patients (16.32%) who had a lateral meniscal tear, and Group C consisted of 92 controls (48.42%). Among the medial-tear patients, 51 (76.12%) were male and 16 (23.88%) were female; among the lateral-tear patients, 24 (77.42%) were male and seven (22.58%) were female; in the control group, 50 (54.35%) were male and 42 (45.65%) were female. The between-group difference in sex distribution was statistically significant (*p* = 0.006), which was subsequently addressed by adjusting for sex in the logistic regression models. Side distribution did not differ between groups (*p* = 0.325), and the mean age was comparable across Group A (23.30 ± 3.73 years), Group B (22.13 ± 3.95 years) and Group C (23.52 ± 3.84 years) (*p* = 0.211).

Both the MPTS and the LPTS differed significantly among the three groups (ANOVA, *p* = 0.001). Groups A and B showed significantly higher medial and lateral slope angles than the control group (Table 2). Within Group A, the MPTS was significantly greater than the LPTS (*p* < 0.05), whereas within Group B, the medial and lateral slope angles did not differ significantly (*p* > 0.05). The corresponding mean differences, 95% CIs and effect sizes for each group–control comparison are given in Table 3; all four comparisons showed medium-to-large effect sizes (Cohen’s d: 0.57–0.90).

**Figure 3:**
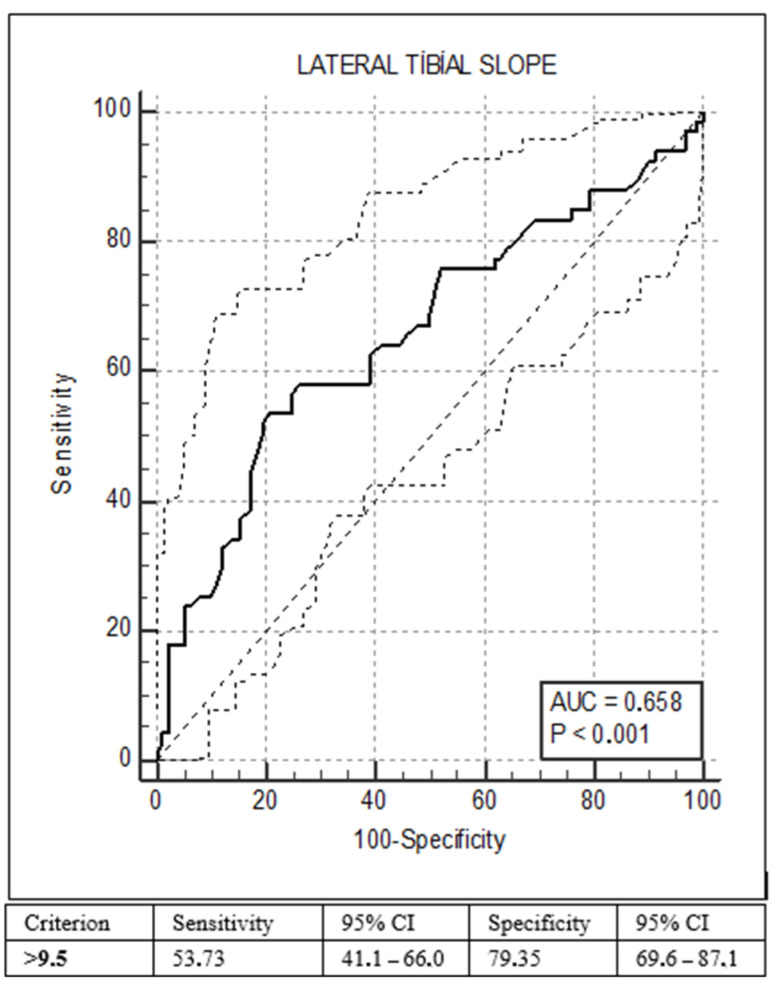
Receiver operating characteristic (ROC) curve for the lateral posterior tibial slope (LPTS) in discriminating patients with an isolated medial meniscal tear (Group A, n = 67) from symptomatic controls (Group C, n = 92). Sensitivity (%) is plotted on the vertical axis against 100 − specificity (%) on the horizontal axis; the diagonal line represents the line of no discrimination (AUC = 0.50). The optimal cut-off value, determined with the Youden index, was 9.5° and is indicated on the curve (sensitivity 53.73%, specificity 79.35%). The area under the curve (AUC) was 0.658 (95% CI 0.57–0.75; *p* < 0.001), indicating modest discriminative ability: the threshold is comparatively specific but insensitive, and should therefore be regarded as supportive rather than confirmatory. AUC, area under the curve; CI, confidence interval; LPTS, lateral posterior tibial slope; ROC, receiver operating characteristic.

**Figure 4:**
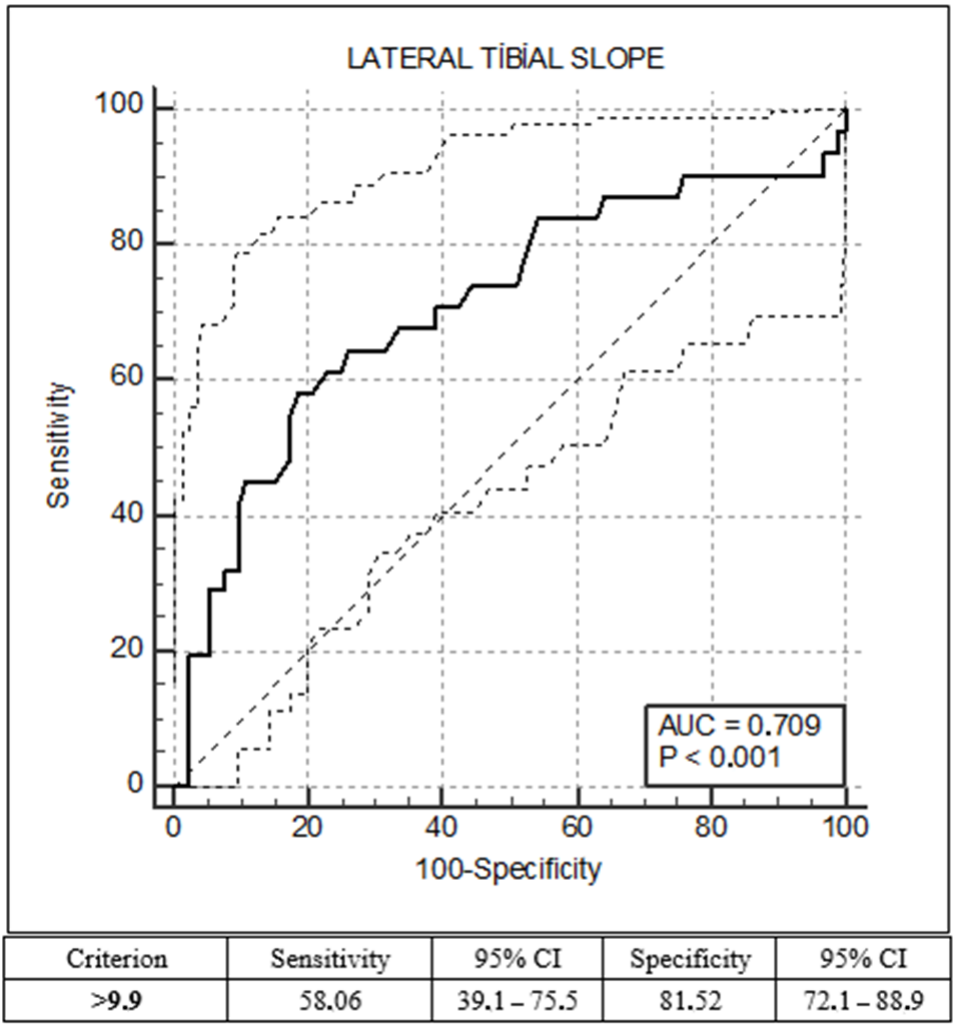
Receiver operating characteristic (ROC) curve for the lateral posterior tibial slope (LPTS) in discriminating patients with an isolated lateral meniscal tear (Group B, n = 31) from symptomatic controls (Group C, n = 92). Axes and reference diagonal are as described in Figure 3. The optimal cut-off value, determined with the Youden index, was 9.9° (sensitivity 58.06%, specificity 81.52%). The area under the curve (AUC) was 0.709 (95% CI 0.60–0.82; *p* < 0.001). As in Figure 3, specificity exceeds sensitivity, so an LPTS above this threshold supports but does not establish the diagnosis. AUC, area under the curve; CI, confidence interval; LPTS, lateral posterior tibial slope; ROC, receiver operating characteristic.

The LPTS of medial-tear patients versus controls yielded a cut-off of 9.5° (Youden index; sensitivity: 53.73%, specificity: 79.35%; AUC: 0.658, 95% CI: 0.57–0.75; *p* < 0.001) (Figure 3). The LPTS of lateral-tear patients versus controls yielded a cut-off of 9.9° (sensitivity: 58.06%, specificity: 81.52%; AUC: 0.709, 95% CI: 0.60–0.82; *p* < 0.001) (Figure 4). In keeping with the modest sensitivities, these thresholds showed high specificity but limited sensitivity and should therefore be interpreted as supportive rather than confirmatory.

**Figure 5:**
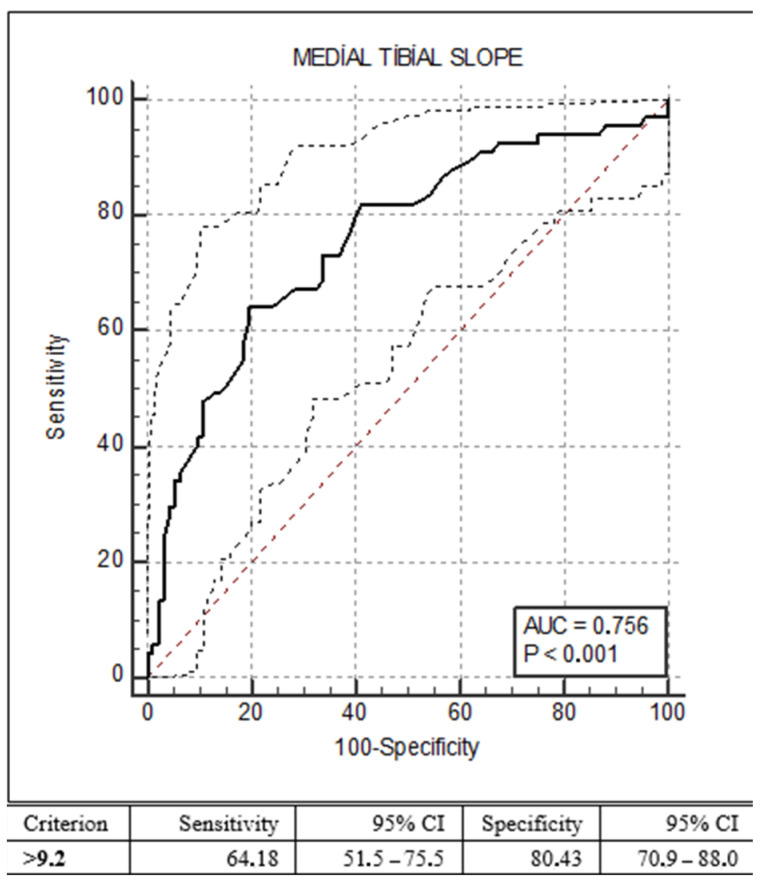
Receiver operating characteristic (ROC) curve for the medial posterior tibial slope (MPTS) in discriminating patients with an isolated medial meniscal tear (Group A, n = 67) from symptomatic controls (Group C, n = 92). Axes and reference diagonal are as described in Figure 3. The optimal cut-off value, determined with the Youden index, was 9.2° (sensitivity 64.18%, specificity 80.43%). The area under the curve (AUC) was 0.756 (95% CI 0.68–0.83; *p* < 0.001), the highest discriminative performance observed among the four ROC analyses reported in this study. AUC, area under the curve; CI, confidence interval; MPTS, medial posterior tibial slope; ROC, receiver operating characteristic.

**Figure 6:**
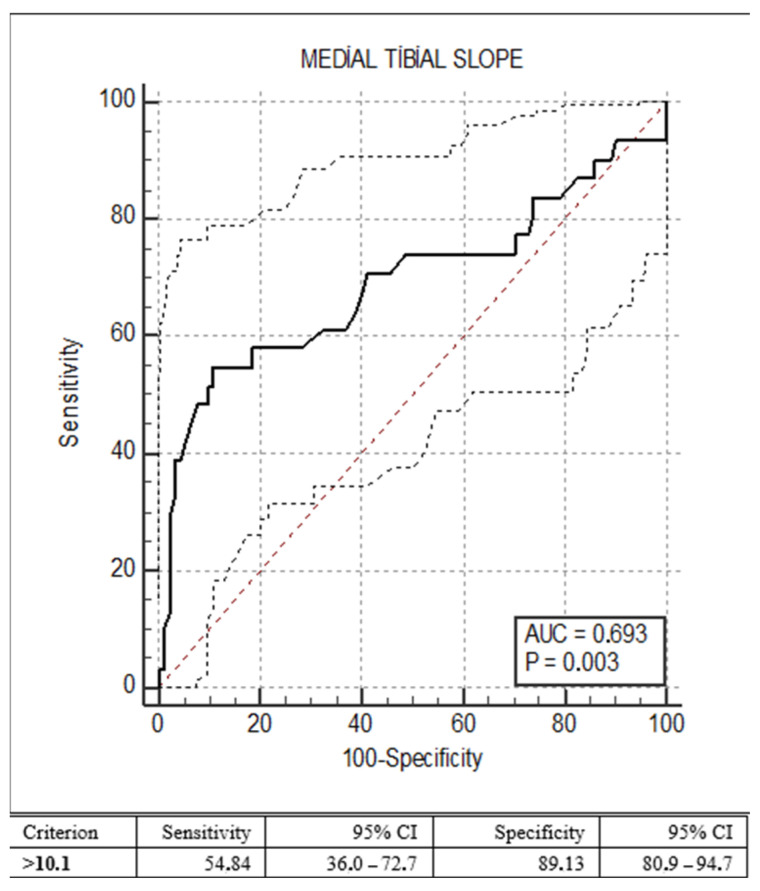
Receiver operating characteristic (ROC) curve for the medial posterior tibial slope (MPTS) in discriminating patients with an isolated lateral meniscal tear (Group B, n = 31) from symptomatic controls (Group C, n = 92). Axes and reference diagonal are as described in Figure 3. The optimal cut-off value, determined with the Youden index, was 10.1° (sensitivity 54.84%, specificity 89.13%). The area under the curve (AUC) was 0.693 (95% CI 0.58–0.81; *p* = 0.003). This threshold shows the highest specificity but a correspondingly low sensitivity, indicating that an elevated MPTS supports, but does not establish, the diagnosis of a meniscal tear. AUC, area under the curve; CI, confidence interval; MPTS, medial posterior tibial slope; ROC, receiver operating characteristic.

The MPTS of medial-tear patients versus controls produced a cut-off of 9.2° (sensitivity: 64.18%, specificity: 80.43%; AUC: 0.756, 95% CI: 0.68–0.83; *p* < 0.001) (Figure 5). The MPTS of lateral-tear patients versus controls produced a cut-off of 10.1° (sensitivity: 54.84%, specificity: 89.13%; AUC: 0.693, 95% CI: 0.58–0.81; *p* = 0.003) (Figure 6). Again, the high specificity but low-to-moderate sensitivity of these cut-offs indicates that an elevated MPTS supports, but does not establish, a diagnosis of a meniscal tear.

To determine whether posterior tibial slope was independently associated with meniscal tears after adjustment for the imbalanced sex distribution, multivariable binary logistic regression analysis was performed (Table 4). After adjustment for age, sex and side, increased MPTS remained independently associated with medial meniscal tears (adjusted OR: 1.36 per 1° increase, 95% CI: 1.19–1.55; *p* < 0.001). Likewise, increased LPTS remained independently associated with lateral meniscal tears (adjusted OR: 1.25 per 1° increase, 95% CI: 1.08–1.44; *p* = 0.003). Male sex was independently associated with medial meniscal tears (OR: 2.08, 95% CI: 1.01–4.30; *p* = 0.047), whereas its association with lateral meniscal tears did not reach significance (OR: 2.47, 95% CI: 0.96–6.37; *p* = 0.061); age and side were not independently associated with either outcome.

## 4. Discussion

The principal finding of this study is that an increased posterior tibial slope is associated with meniscal tears in young adults. These results are consistent with previous work in adult populations [21]. Moon et al. reported that the posterior tibial slope is strongly associated with medial meniscal root tears without significantly influencing the tear pattern. In our cohort, the MPTS cut-off for medial meniscal tears was 9.2° (sensitivity: 64.18%, specificity: 80.43%).

Biomechanical studies show that an increased tibial slope produces a linear relationship between knee loading and anterior tibial translation. This forward translation raises the shear forces acting on the ACL and the menisci, increasing the risk of injury to these structures [11,12,22,23]. An increased posterior tibial slope may therefore facilitate anterior tibial displacement and create a mechanical environment that favours meniscal injury; however, because the present design is observational, this mechanism should be regarded as a plausible explanation rather than a demonstrated cause. The wider value, and the limits, of quantifying mechanical parameters in orthopaedic practice are illustrated by the arthroplasty literature. The tibial slope is itself a parameter that is deliberately set and controlled during total knee arthroplasty, where its optimisation, together with posterior condylar offset, governs condylar roll-back and post-operative knee kinematics [24]. In a systematic review and meta-analysis of 116 studies comprising 6869 participants, Becker et al. pooled the distraction force applied during tension-controlled ligament-balanced total knee arthroplasty and derived reference values of 149.9 N at 0° of extension and 139.5 N at 90° of flexion, with values that were consistent across native, cadaveric and computer-model knees [25]. This synthesis demonstrates that a measurable mechanical parameter can be distilled into a clinically usable reference value and that doing so requires large, pooled and methodologically homogeneous datasets. By the same standard, the single-centre, radiographic slope thresholds reported below should be regarded as preliminary morphological reference points rather than as validated decision limits.

Several studies report a significant relationship between the posterior tibial slope and meniscal tears [14,17,21]. Kolbe et al. emphasised that a steep lateral slope is a risk factor for posterolateral meniscal root tears, and Moon et al. observed a higher slope in knees with medial meniscal root tears.

Alici et al. measured the tibial slope on both radiographs and MRI in 212 knees and found a significantly higher LPTS in the lateral-tear group than in controls, but no significant difference in the MPTS between medial-tear patients and controls. In our study, both the MPTS and the LPTS were significantly higher in the tear groups than in the control group. The relevant cut-offs were 9.2° for the MPTS in the medial-tear group and 9.9° for the LPTS in the lateral-tear group. A distinguishing feature of the present study is that all tears were directly visualised at arthroscopy, which confirmed the presence and location of the meniscal lesion and thereby strengthened the diagnostic certainty of the tear classification. However, it should be emphasised that the slope itself was measured only radiographically; arthroscopy was used to verify the tear but not to validate the radiographic measurement or establish any causal link. Increased participation in athletic activity and high-performance demands, particularly in younger patients, may heighten the predisposition to meniscal tears in individuals with a high posterior tibial slope [26].

Unlike the MRI-based measurements of Alici et al., a primary aim of our study was to use plain radiography—a more economical and accessible modality—to identify a morphological risk marker, thereby reducing reliance on MRI and improving practicality, cost and accessibility in routine practice.

Optimal thresholds were determined by ROC analysis using the Youden index. The MPTS threshold in the medial-tear group was 9.2° (sensitivity: 64.18%, specificity: 80.43%) and the LPTS threshold in the lateral-tear group was 9.9° (sensitivity: 58.06%, specificity: 81.52%). The corresponding areas under the curve were modest (0.756 and 0.709, respectively), indicating fair rather than strong discrimination and reinforcing that these thresholds are supportive rather than confirmatory. Thus, the probability of a medial tear was higher in individuals with an MPTS above 9.2° and that of a lateral tear was higher with an LPTS above 9.9°. Given the modest sensitivity of these thresholds, they are best interpreted as supportive morphological markers rather than stand-alone diagnostic indicators. This point warrants emphasis. Across the four ROC models, the areas under the curve ranged from 0.693 to 0.756, which corresponds to fair discrimination only; a substantial overlap in slope values therefore persists between knees with and without a meniscal tear. At the Youden-derived cut-offs, specificity was acceptable (80.4–89.1%) but sensitivity was low (54.8–64.2%), meaning that more than one-third of arthroscopically confirmed tears occurred in knees with a slope below the threshold. In practical terms, a slope above the cut-off modestly increases the probability that knee pain in a young adult is attributable to a meniscal tear, whereas a slope below the cut-off carries no reassurance and cannot be used to withhold further imaging. These values should therefore not be adopted as diagnostic, screening or triage thresholds, nor used to justify surgical decision-making; they are best understood as supportive morphological markers that add context to the history, physical examination and MRI findings. External validation in independent, prospectively recruited cohorts—preferably incorporating the slope into multivariable risk models alongside activity level, BMI and sex rather than treating it as an isolated dichotomised variable—is required before any threshold-based clinical application can be recommended.

Denk et al. reported an MPTS threshold of 10.3° (sensitivity: 73.3%, specificity: 78.9%) in isolated medial meniscal tears [19], whereas Moon et al. reported a slope threshold of 6.6° [21]; these differences are attributable to variations in patient selection. Dare et al. observed that the posterior tibial slope tends to decrease slightly with age [16]. The comparatively high thresholds in our study likely reflect our young cohort (18–30 years) and align closely with those of Denk et al., who studied a similar age group.

There is no consensus regarding differences between medial and lateral slopes. Li et al. found no significant medial–lateral difference on MRI [18], whereas Stijak et al. reported a greater lateral slope in ACL-injured knees and a greater medial slope in the controls with patellofemoral pain [27]. In our cohort, a significant medial–lateral difference was observed in medial-tear patients, which may be clinically relevant. Kim et al. detected medial meniscal ramp lesions in 42% of ACL-reconstruction patients and reported a higher slope in these patients (8.3 ± 1.7° vs. 6.9 ± 2.4° in controls) [13].

In our study, the MPTS was 10.11 ± 3.23° in medial-tear patients and 10.11 ± 4.23° in lateral-tear patients, and the LPTS was 9.42 ± 3.67° and 9.79 ± 3.55°, respectively, versus 7.50 ± 2.63° (MPTS) and 7.60 ± 2.83° (LPTS) in the controls, consistent with the values reported in Table 2. Because ramp lesions are peripheral meniscal tears, these findings are in line with the literature.

Bernholt et al. reported an LPTS of 9.1° and an MPTS of 7° in patients with lateral meniscal root tears among 103 patients and controls, without significant differences in lateral femoral condyle depth/width, lateral tibial plateau depth or subluxation [28]; like us, they analysed the medial and lateral slopes separately. Conversely, some studies suggest that the slope is not decisive: Alici et al., using MRI and the proximal tibial anatomical axis, found no significant between-group difference in the medial slope (3.18°, 3.64°, 3.00° and 3.27° across their four groups) [17], which contrasts with our findings. Studies using comparable methods report higher healthy-population values (6.8 ± 1.8° to 7.7 ± 2.4°) than those of Alici et al. [29,30], a discrepancy likely explained by differences in measurement method, reference-axis selection and population characteristics.

In previous studies, the slope was measured on both MRI and plain radiography. Alici et al., Kim et al. and Bernholt et al. used MRI to evaluate the medial and lateral angles separately [13,17,28]. Here, we used plain radiography; although MRI is considered more reliable, standing lateral radiographs were preferred because of their wider availability, lower cost, shorter evaluation time and larger field of view. Different radiographic techniques for slope measurement have been reported to yield similar results [31].

A further consideration specific to the age band studied here is physical activity. Participation in pivoting, cutting and contact sports peaks between 18 and 30 years, and cumulative athletic exposure is itself among the strongest determinants of meniscal injury risk in this population [3,26]. Because sport type, competitive level and training volume were not recorded, we cannot exclude the possibility that the tear groups were, on average, more athletically active than the symptomatic controls, who were investigated for knee pain of non-meniscal origin. The consequences for our estimates depend on how activity relates to slope. If athletic exposure is distributed independently of tibial morphology, it would behave as a competing, unmeasured risk factor: it would add unexplained variance and reduce precision, but would not be expected to bias the slope–tear association systematically. If, conversely, individuals with a steeper posterior tibial slope preferentially select into or persist in high-demand pivoting sports, or if a steep slope becomes injurious only once a threshold of repetitive loading is exceeded, then activity would lie on the causal pathway or act as an effect modifier, and the adjusted odds ratios reported here (1.36 per 1° for MPTS and 1.25 per 1° for LPTS) would in part reflect athletic exposure rather than bony morphology alone. A third possibility works in the opposite direction: highly active control participants with a steep slope who have not yet sustained a tear would attenuate rather than inflate the observed association. Because the direction and magnitude of this residual confounding cannot be determined from the present data, the odds ratios should be read as unadjusted for exposure. The relatively narrow age range, the exclusion of ligament injury and the use of symptomatic rather than asymptomatic controls—who were, by definition, sufficiently symptomatic to undergo MRI and are therefore unlikely to represent a wholly sedentary comparison group—mitigate but do not eliminate this concern. Prospective studies that prospectively capture sport type, competitive level, weekly training hours and cumulative exposure, alongside BMI, are needed to establish whether the posterior tibial slope contributes to meniscal injury risk independently of activity and whether its effect is amplified in athletes; such data would also clarify whether slope-specific thresholds differ between athletic and non-athletic young adults.

### Limitations

This study has several limitations that temper its conclusions. First, the design is retrospective and observational, so the associations reported cannot establish causation. Second, the sex distribution differed significantly between groups; although we adjusted for age, sex and side in the multivariable analysis, residual confounding cannot be excluded. Third, BMI and sport/activity data—both of which may influence the slope and the risk of meniscal tears—were unavailable, so the findings should be regarded as associational and subject to residual confounding. This limitation is of particular importance in an 18–30-year-old cohort, in whom athletic exposure is a principal driver of meniscal injury; as detailed above, the direction of the resulting bias cannot be established, and residual confounding by activity level may have either inflated or attenuated the reported odds ratios. Fourth, the slope was measured on plain radiographs only and was not cross-validated against MRI or CT, which some authors consider more accurate [31,32]. Measurement reliability was addressed by triplicate readings and by inter- and intraobserver ICC analysis; nonetheless, the single-modality radiographic measurement remains a limitation. In future work, we recommend confirming radiographic slope measurements with MRI in larger prospective cohorts using multiple independent observers, and collecting BMI and activity data.

## 5. Conclusions

In this retrospective cohort of young adults, an increased posterior tibial slope was associated with isolated meniscal tears: both the medial and lateral slope angles were significantly higher in patients with arthroscopically confirmed tears than in symptomatic controls. A medial slope above 9.2° was associated with medial meniscal tears and a lateral slope above 9.9° was associated with lateral meniscal tears, suggesting a side-specific morphological relationship. Because the analysis is observational, lacks BMI and activity data, and shows only modest sensitivity, these results should be interpreted as evidence of an association rather than causation. With that caveat, the posterior tibial slope—which can be readily measured on standard lateral radiographs—could serve as an accessible supportive marker in the clinical evaluation and risk stratification of young patients with suspected meniscal pathology, ideally combined with clinical findings and MRI rather than used in isolation. It must be stressed, however, that the discrimination achieved was only fair (AUC 0.693–0.756) and that sensitivity did not exceed 64.2%. The cut-offs of 9.2° and 9.9° are therefore proposed as supportive morphological reference points for research and risk stratification, and explicitly not as clinically applicable decision thresholds: they should not be used to rule a meniscal tear in or out, to select patients for arthroscopy, or to substitute for MRI. Prospective external validation, together with the collection of activity and BMI data, is required before any clinical application can be considered.

## Figures and Tables

**Table 1:** Demographic and clinical characteristics of the study groups.

Characteristic	Medial Tear (n = 67)	Lateral Tear (n = 31)	Control (n = 92)	*p*
Age, years (mean ± SD)	23.30 ± 3.73	22.13 ± 3.95	23.52 ± 3.84	0.211
Sex, male/female (n)	51/16	24/7	50/42	0.006
Side, right/left (n)	30/37	15/16	52/40	0.325
BMI, kg/m^2^ (mean ± SD)	NA	NA	NA	–

Data are shown as the mean ± SD or n. Age and side did not differ significantly between groups, whereas the sex distribution did (*p* = 0.006); sex was therefore adjusted for in the multivariable analysis. BMI was not recorded in the source records and is reported as not available (NA); this is acknowledged as a limitation.

**Table 2:** Comparison of medial (MPTS) and lateral (LPTS) posterior tibial slope angles of groups (one-way ANOVA).

Variable	Medial Tear (Mean ± SD)	Lateral Tear (Mean ± SD)	Control (Mean ± SD)	*p*
MPTS (°)	10.11 ± 3.23 ^a^	10.11 ± 4.23 ^a^	7.50 ± 2.63 ^b^	0.001 *
LPTS (°)	9.42 ± 3.67 ^a^	9.79 ± 3.55 ^a^	7.60 ± 2.83 ^b^	0.001 *

MPTS, medial posterior tibial slope; LPTS, lateral posterior tibial slope. Superscripts ^a^ and ^b^ denote homogeneous subsets based on Tukey HSD; groups with the same letter do not differ significantly. * *p* < 0.05.

**Table 3:** Pairwise comparison of posterior tibial slope angles between each tear group and the control group.

Comparison (vs. Control)	Mean Diff. (°)	95% CI	Cohen’s d
MPTS—medial tear	2.61	1.67 to 3.55	0.90
MPTS—lateral tear	2.61	1.03 to 4.19	0.84
LPTS—medial tear	1.82	0.77 to 2.87	0.57
LPTS—lateral tear	2.19	0.81 to 3.57	0.72

Effect sizes were derived from the group means, standard deviations and sample sizes reported in Table 2. A Cohen’s d of 0.5–0.8 indicates a medium effect and ≥0.8 indicates a large effect.

**Table 4:** Multivariable binary logistic regression for the association between posterior tibial slope and meniscal tears (adjusted for age, sex and side).

Variable	Adjusted OR	95% CI	*p*
**Model 1—medial tear vs. control**			
MPTS (per 1°)	1.36	1.19–1.55	<0.001
Age (per year)	0.98	0.90–1.06	0.603
Sex (male vs. female)	2.08	1.01–4.30	0.047
Side (right vs. left)	0.68	0.35–1.31	0.246
**Model 2—lateral tear vs. control**			
LPTS (per 1°)	1.25	1.08–1.44	0.003
Age (per year)	0.92	0.82–1.03	0.151
Sex (male vs. female)	2.47	0.96–6.37	0.061
Side (right vs. left)	0.76	0.33–1.74	0.514

OR, odds ratio; CI, confidence interval. Each model was adjusted for age, sex and side; bold sub-headings denote the two regression models.

## Data Availability

The data are available from the corresponding author upon reasonable request, subject to privacy and ethical restrictions.
